# Predictors of giant cell arteritis in patients with polymyalgia rheumatica in southern Sweden—a retrospective study

**DOI:** 10.1093/rap/rkaf112

**Published:** 2025-09-29

**Authors:** Charlotta Fors, Ulf Bergström, Aladdin J Mohammad, Carl Turesson

**Affiliations:** Rheumatology, Department of Clinical Sciences, Lund University, Malmö, Sweden; Department of Rheumatology, Skåne University Hospital, Malmö, Sweden; Rheumatology, Department of Clinical Sciences, Lund University, Malmö, Sweden; Rheumatology, Department of Clinical Sciences, Lund University, Lund, Sweden; Department of Rheumatology, Skåne University Hospital, Lund, Sweden; Department of Medicine, University of Cambridge, Cambridge, UK; Rheumatology, Department of Clinical Sciences, Lund University, Malmö, Sweden; Department of Rheumatology, Skåne University Hospital, Malmö, Sweden

**Keywords:** epidemiology, polymyalgia rheumatica, giant cell arteritis, inflammation, primary care rheumatology

## Abstract

**Objective:**

To identify predictors of GCA in patients with PMR.

**Methods:**

Patients with PMR were identified among participants in two population-based health surveys. Those with a registered diagnosis indicating PMR in national and regional registers (the latter including primary care) were included. Medical records from hospitals and primary care were systematically reviewed. PMR diagnoses were verified by a rheumatologist in an independent review. Potential predictors were examined using logistic regression analysis.

**Results:**

Of 1508 medical records, 1030 had sufficient data available. PMR diagnoses were verified in 626 patients (61%). GCA was diagnosed within 1 month of PMR diagnosis in 37 patients and at a later time point in 20 patients. Female patients were more likely to develop GCA at PMR diagnosis or later [odds ratio (OR) 2.38 (95% CI 1.23, 4.61)]. Higher ESR and CRP levels were also associated with GCA. A lower risk for GCA was seen in those presenting with pain/stiffness in the hip [OR 0.51 (95% CI 0.28, 0.92)].

**Conclusion:**

In this large cohort of patients with verified PMR, GCA was diagnosed in a limited subset (9%) and was more common in females. A lower risk for GCA was seen in patients with pain/stiffness in the hip at onset of PMR, suggesting that prominent musculoskeletal symptoms and cranial arteritis represent different parts of the GCA–PMR spectrum.

Key messages

- Of 626 patients with verified PMR, 9% developed GCA at PMR diagnosis or later.
- GCA was less common in patients with pain/stiffness in the hip at the onset of PMR.

## Introduction

PMR is an inflammatory condition that typically presents with a subacute to acute onset of pain and morning stiffness in the neck, shoulders, upper arms and pelvic girdle together with elevated acute phase reactants. PMR can occur independently but sometimes coexists with GCA, the most commonly occurring vasculitis in Europe and North America [1]. GCA is characterized by vascular wall inflammation of medium and large vessels. The temporal artery or other branches of the carotid artery are typically afflicted, but manifestations in the aorta, large supra-aortic vessels or their branches can also occur. PMR and GCA almost exclusively occur in individuals ≥50 years of age and are described to be two to three times more common in women [2, 3]. Both PMR and GCA have the highest incidence in populations of northern European, particularly Scandinavian, origin. The incidence of PMR and GCA in these areas is reported to be 34–113 and 15–44 cases per 100 000, respectively, in individuals ≥50 years of age [4].

The coexistence of PMR and GCA is well studied but the nature of the relationship is not fully clarified. Several characteristics overlap, such as elevated acute phase reactants (ESR and CRP), weight loss and other constitutional symptoms. Systemic inflammation with a predominant IL-6 signature and expansion of IL-17-producing T cells has been seen in both PMR and GCA [5, 6]. IL-6 is important in both PMR and GCA, whereas the IL-12/INF-γ pathway stimulating differentiation of Th-1 cells is a feature distinctive for GCA [7, 8]. In a meta-analysis of 13 published studies, findings consistent with subclinical GCA were present in 23% of patients with PMR screened using temporal artery biopsy (TAB) or imaging [9]. A 4-fold higher relapse rate has been described if patients with PMR also had subclinical GCA, defined as the presence of halo sign on ultrasound of the temporal, axillary or subclavian arteries [10]. Between 40 and 60% of patients with GCA present with clinical manifestations of PMR [11]. It has been suggested that clinically isolated PMR with bursitis and/or periarthritis, cranial GCA, large vessel GCA and polymyalgia with peripheral arthritis, including remitting seronegative symmetrical synovitis with pitting oedema should be considered under a common term of GCA–PMR spectrum disease [5, 6].

The diagnoses of PMR and GCA are both based on clinical manifestations and elevated acute phase reactants. In addition, different imaging techniques can contribute valuable information [12, 13]. TAB has long been a standard diagnostic tool for GCA, whereas in later years the use of vascular ultrasound has become more widespread [13]. Several different sets of classification criteria have been proposed for PMR [14–19] and GCA [13, 20, 21]. The criteria are constructed primarily for research purposes but are sometimes used for diagnostic guidance in clinical practice. An international multicentre study surveying general practitioners (GPs) and rheumatologists found that a median of 25% of patients with PMR were referred to specialist care [22]. A higher starting dose of prednisolone (>25 mg/day) was more often prescribed by GPs in primary healthcare (30%) than by rheumatologists (12%) [22].

In Sweden and in many other countries, PMR is typically managed in primary healthcare while patients with GCA are often followed at a rheumatology clinic. It is recommended that patients with suspected large vessel vasculitis should be urgently referred to a specialist team for investigation [23]. Patients with GCA are at risk of severe complications, including permanent loss of vision, caused by anterior ischaemic optic neuropathy, cerebrovascular events, infarction of the tongue and aortic aneurysm or other large vessel involvement [6]. Early initiation of treatment is therefore crucial. Improved knowledge about predictors of GCA in patients with PMR could be valuable for identifying individuals at higher risk for GCA and enable risk-stratified management. The objective of this study was to identify risk factors for GCA in patients with PMR and thereby provide useful information for the management of patients with PMR.

## Methods

### Source population

Patients with PMR were identified among participants in two population-based prospective health surveys of residents in Malmö, Sweden. These surveys included physical examination, blood samples and patient-reported questionnaires on lifestyle factors, level of education and reproductive factors.

For the Malmö Diet and Cancer Study (MDCS) [24], all women born between 1923 and 1950 and all men born between 1923 and 1945 were invited. Exclusion criteria were inadequate Swedish language skills and mental incapacity. With a participation rate of 41%, a total of 12 121 men and 19 326 women joined the study at baseline between 1991 and 1996. Of these, 28 098 individuals completed all baseline examinations.

The Malmö Preventive Medicine Program (MPMP) [25] recruited 22 444 men (born between 1921 and 1949) and 10 902 women (born between 1925 and 1938) in Malmö between 1974 and 1992, with an overall response rate of 71%.

### Retrieval of patients diagnosed with PMR

The Swedish National Patient Register (NPR) contains statistics of diseases for all inpatient care in Sweden since 1987 and for specialized outpatient care since 2001. Data reporting to the NPR is virtually complete for inpatient care and coverage has gradually improved for specialized outpatient care [26]. The Skåne Healthcare Register (SHR) is a regional database that contains information from electronic medical records and administrative databases on healthcare consultations in the Skåne region from since 1998. Healthcare consultations to all types of healthcare professionals in inpatient care, specialized outpatient care and primary care generate data entries that are automatically transferred to the SHR. Proportions of consultations with an assigned International Classification of Diseases (ICD) code have been ≥80% in public primary care after 2004 and nearly complete for inpatient care and secondary outpatient care during the same time period [27]. Diagnostic codes from private care are not transferred to the SHR.

Participants from either the MDCS or the MPMP with a subsequent diagnosis of PMR (ICD-10 code M353) or GCA with PMR (ICD-10 code M315) in the SHR were identified. Further, MDCS participants with a diagnosis of PMR or GCA with PMR in the NPR were also identified. Medical records were obtained from regional hospital medical records systems (Melior, Sieview) for electronic access. Further, complete medical records through December 2018 were obtained from the regional primary healthcare system [Profdoc Medical Office (PMO)].

### Data collection

All the medical records from primary healthcare (PMO) and inpatient care (regional hospital computerized system; Melior/Sieview) were systematically reviewed when available. A data collection sheet was completed for the patients with available relevant data. The patients were excluded if medical records with information on first-time onset of PMR were unavailable or if the information was insufficient for the data collection. An independent review was performed by an experienced rheumatologist (C.T.) for verification of the PMR diagnosis. For a subset of patients (*n* = 81), an additional review for verification of a PMR diagnosis was performed by two rheumatologists (C.T. and A.J.M.) and one experienced GP (U.B.). The patients with verified PMR were classified as PMR or PMR with GCA, based on the records review. Furthermore, the patients were classified according to international classification criteria for PMR [14, 17, 18] and for GCA [20, 28]. The date of first clinical diagnosis of PMR and GCA was determined based on a review of case records.

### Statistics

Descriptive data for baseline characteristics for all patients and by subset (PMR with no GCA, PMR with GCA at diagnosis or at any time after that, PMR with GCA >1 month after PMR diagnosis) are presented as number (%), mean (s.d.) or median [interquartile range (IQR)], as appropriate. Normality of data distribution was assessed based on graphic evaluation and also using the Shapiro–Wilk test. Logistic regression was performed to determine possible factors associated with GCA at the PMR diagnosis or later. In order to assess predictors of a late GCA diagnosis, separate analyses were performed including only those with a documented diagnosis of GCA >1 month after the first PMR diagnosis. The cut-off of 1 month was based on expert opinion and was considered to separate those diagnosed with GCA during workup for PMR from those with a later diagnosis.

Pearson’s test or Spearman’s test was used to test for collinearity prior to selection of variables for the multivariate analysis. A correlation coefficient >0.3 in a significant correlation (*P* < 0.05) was considered to indicate collinearity. The relation between different characteristics at the PMR diagnosis and time to GCA was estimated using Cox regression. Variables with numbers of ≤5 were not included in the regression analyses.

### Ethics

The study was approved by the regional research ethics committee for southern Sweden (registration number 2018/875, date of approval 22 November 2018). Participants in the MPMP and the MDCS gave their written informed consent for use of their data for research. Individual consent for the present study was not required by the research ethics committee.

## Results

### Patient disposition

**Figure 1.**
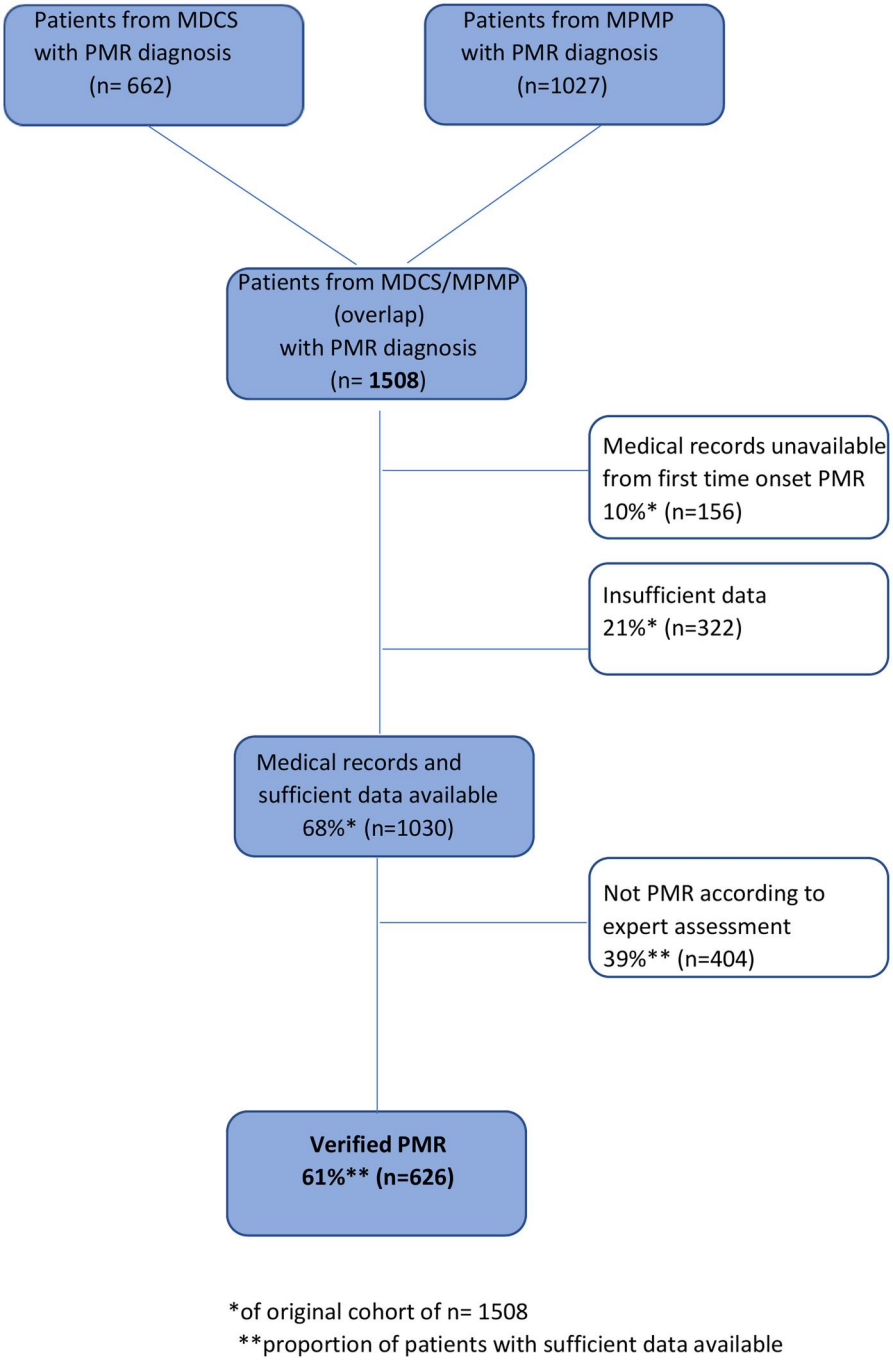
Identification and selection of patients with PMR

A total of 1508 patient records from cases with a registered diagnosis of PMR were subjected to a structured review. Of these, 156 had missing information in the medical records regarding the first episode of PMR. An additional 322 records were considered to have an insufficient amount of information available for validating the diagnosis and completing the form, leading to exclusion of a total of 478 patients (Fig. 1).

### Validity of PMR diagnoses

Of the 1030 patients with sufficient available information for completing the form, the PMR diagnosis was in agreement with the independent expert review in 626 cases (61%) (Fig. 1). The level of agreement on diagnosis was 80% among the three investigators that performed an additional review of a subset of patients (*n* = 81).

### Patient characteristics at PMR diagnosis

The mean age at PMR diagnosis was 75.8 years (s.d. 6.8). Among the patients with verified PMR, 44% (*n* = 278) fulfilled the ACR/EULAR criteria [18], 83% (*n* = 521) the Bird criteria [14] and 57% (*n* = 359) the Healey criteria [17]. A total of 89% (*n* = 554) fulfilled one or more of these criteria sets. The median symptom duration at PMR diagnosis was 30 days (IQR 14–49). Nearly all patients with a validated diagnosis of PMR had elevated ESR or CRP levels (Table 1).

**Table 1.** Baseline characteristics at PMR diagnosis, overall and stratified by GCA status.

Characteristics	All	PMR only	GCA at or within 1 month of PMR diagnosis	GCA >1 month after PMR diagnosis
Patients, *n*^a^	626	566	37	20
Duration of symptoms, days, median (IQR)	30 (14–49)	30 (14–45)	30 (14–60)	21 (14–30)
Age, years, mean (s.d.)	75.8 (6.8)	75.8 (6.7)	74.3 (6.7)	76.1 (6.6)
Female, *n* (%)	393 (62.8)	346 (61.1)	29 (78)	16 (80)
Elevated ESR or CRP, *n* (%)^b^	612 (98.9)	555 (98.9)	35 (97.2)	19 (100)
ESR, mm/h, mean (s.d.)^b^	61 (25)	60 (25)	74 (26)	63 (17)
CRP, mg/l, median (IQR)^b^	49 (28–85)	46 (27–82)	85 (37–115)	60 (33–116)
RF-positive within tested, *n*/*N* (%)	23/202 (11)	20/189 (11)	0/7 0	2/4 (50)
ACPA positive within tested, *n*/*N* (%)	2/158 (1)	2/145 (1)	0/6 0	0/3 0
Morning stiffness, *n* (%)^b^	250 (83.9)	234 (84.8)	9 (64)	6 (100)
Hip pain or stiffness, *n* (%)	308 (50.5)	288 (51.8)	13 (37)	5 (31)
Rapid response to glucocorticoids, *n* (%)^c^	274 (55.1)	263 (59.4)	1 (3)	8 (50)
Prednisolone dose, mean (s.d.)^d^	23 (11)	22 (9)	46 (16)	21 (6)
Documented depression, *n* (%)^e^	37 (6)	34 (6)	3 (8)	0
Documented weight loss, *n* (%)	90 (15)	77 (14)	11 (31)	1 (5)
Current smoker, *n* (%)	75 (13)	67 (13)	5 (14)	2 (11)
Ever smoker, *n* (%)	326 (62)	300 (62.5)	17 (57)	8 (44)
Years since quit smoking, mean (s.d.)	26 (17)	26 (17)	21 (20)	38 (14)

a Includes three cases with GCA of unclear diagnosis date.

b Missing: elevated ESR or CRP, 7; ESR, 35; CRP, 87; morning stiffness all, 333; morning stiffness PMR only, 295; morning stiffness GCA <1 month of PMR, 23; morning stiffness GCA >1 month of PMR, 14.

c Within 1 week on a starting dose of <21 mg prednisolone.

d Daily dosage (mg) of prednisolone prescribed at onset of PMR.

e Within 6 months of onset of PMR.

### Patients with PMR and GCA

A total of 63 patients also had GCA prior to, at or after the PMR diagnosis. Among those with verified PMR, 37 patients were diagnosed with GCA at or within 1 month of the PMR diagnosis and 20 patients developed GCA >1 month after the PMR diagnosis (Table 1). An additional three patients had a GCA diagnosis >1 month prior to the first note of a clinical diagnosis of PMR and three patients had an unknown date of GCA diagnosis. For the whole cohort of patients with verified PMR, 63% were women, whereas the corresponding percentage was 78% for those with GCA within 1 month of the PMR diagnosis and 80% if GCA had been diagnosed >1 month after the PMR diagnosis (Table 1).

The majority of patients diagnosed with GCA presented with cranial symptoms [e.g. 81% reported headache (Table 1)]. The ACR 1990 classification criteria for GCA [20] were fulfilled in 71% (*n* = 45) of the whole subset of patients with PMR with GCA, in 73% (*n* = 27) of those with GCA within 1 month of the PMR diagnosis and in 75% (*n* = 15) with GCA >1 month after the PMR diagnosis. The corresponding numbers for fulfilment of the ACR/EULAR 2022 GCA criteria [13] were 87% (*n* = 55), 89% (*n* = 33) and 90% (*n* = 18), respectively (Table 2).

**Table 2.** Fulfilment of GCA criteria, overall and by time from PMR to GCA.

Criteria	All GCA (*N* = 63)	GCA at or within 1 month of PMR diagnosis (*n* = 37)	GCA >1 month after PMR diagnosis (*n* = 20)
Fulfilment of 1990 ACR GCA criteria^a^	45 (71)	27 (73)	15 (75)
Age ≥50 years	63 (100)	37 (100)	20 (100)
Localized pain in the head	46 (81)	27 (80)	16 (89)
Temporal artery abnormality	25 (42)	17 (47)	7 (37)
ESR ≥50 mm/h	44 (70)	29 (78)	12 (60)
Abnormal TAB	34 (54)	18 (49)	13 (65)
Fulfilment of ACR/EULAR 2022 GCA criteria^b^	55 (87)	33 (89)	18 (90)
Positive TAB and/or halo sign on US	34 (54)	18 (49)	13 (65)
ESR ≥50 mm/h and/or CRP ≥10 mg/l	56 (89)	35 (95)	17 (85)
Visual symptoms/sight loss	19 (32)	10 (29)	8 (42)
Morning stiffness (shoulders, neck)	17 (27)	9 (24)	6 (30)
Jaw and/or tongue claudication	25 (42)	16 (44)	9 (47)
Scalp tenderness	23 (39)	16 (44)	6 (33)
Bilateral axillary involvement on imaging and/or FDG-PET activity throughout aorta	0	0	0

FDG-PET: fluorodeoxyglucose positron emission tomography.

Data presented as *n* (%).

a Hunder *et al*. [22].

b Ponte C *et al*. [28].

### Predictors of GCA

Female patients were more likely to have or develop GCA [odds ratio (OR) 2.38 (95% CI 1.23, 4.61) for GCA within 1 month before or any time after PMR diagnosis]. Higher ESR, CRP in the highest quartile and higher prednisolone dose at the PMR diagnosis were also associated with GCA (Table 3). There were negative associations with morning stiffness and with pain/stiffness of the hip. In a multivariate analysis, female sex [OR 2.74 (95% CI 1.13, 6.62)] and higher prednisolone dose [OR 2.28 per s.d. (95% CI 1.77, 2.94)] were both associated with a higher risk for GCA, whereas pain/stiffness of the hip was less common in patients with PMR who also developed GCA at the PMR diagnosis or later [OR 0.47 (95% CI 0.23, 0.97)] (Table 3).

**Table 3.** Predictors of GCA at PMR diagnosis or later: logistic regression

Variables	Univariate OR (95% CI)	Multivariate^b^ OR (95% CI)
Symptoms ≥30 days at PMR diagnosis	0.90 (0.50, 1.60)	NI
Age at PMR diagnosis per s.d.^a^	0.88 (0.67, 1.16)	NI
Female	**2.38 (1.23, 4.61)**	**2.74 (1.13, 6.62)**
ESR at PMR diagnosis per s.d.^a^	**1.47 (1.11, 1.95)**	1.28 (0.90, 1.83)
CRP at PMR diagnosis (mg/l)		
Quartile 1 (0–27)	1.00 (ref)	NI
Quartile 2 (27.01–48)	0.63 (0.22, 1.82)	NI
Quartile 3 (48.01–84)	1.32 (0.54, 3.25)	NI
Quartile 4 (≥84.01)	**2.52 (1.11, 5.73)**	NI
Morning stiffness	0.45 (0.16, 1.27)	NI
Hip pain or stiffness	**0.51 (0.28, 0.92)**	**0.47 (0.23, 0.97)**
Prednisolone dose per s.d.^a^^,^^c^	**2.32 (1.82, 2.96)**	**2.28 (1.77, 2.94)**
Documented depression^d^	0.87 (0.26, 2.94)	NI
Documented weight loss	1.71 (0.86, 3.39)	NI
Smoker at PMR diagnosis	1.00 (0.43, 2.29)	NI
Ever smoker	0.65 (0.36, 1.18)	NI

ref: reference; NI: not included.

Significant associations are in bold.

a s.d.: age 6.7 years, ESR 24.6 mm/h, prednisolone dose 10.7 mg.

b Includes all variables in the column.

c Daily dosage (mg) of prednisolone prescribed at the onset of PMR.

d Within 6 months of onset of PMR.

There were no statistically significant baseline predictors of development of GCA >1 month after the PMR diagnosis (Table 4) but there was a trend towards an association with female sex [OR 2.54 (95% CI 0.84, 7.71)] and towards a negative association with pain/stiffness of the hip [OR 0.42 (95% CI 0.15, 1.23)]. A similar pattern was observed in a Cox regression model of the relation between female sex and time to GCA diagnosed >1 month after the PMR diagnosis [hazard ratio (HR) 2.43 (95% CI 0.81, 7.28)] but there were no statistically significant associations between baseline characteristics and time to later GCA (Table 5).

**Table 4.** Predictors of GCA >1 month after PMR diagnosis: logistic regression

Variables	OR	95% CI
Symptoms ≥30 days at PMR diagnosis	0.42	0.15, 1.23
Age at PMR diagnosis per s.d.^a^	1.06	0.67, 1.66
Female	2.54	0.84, 7.71
ESR at PMR diagnosis per s.d.^a^	1.10	0.69, 1.74
CRP at diagnosis (mg/l)		
Quartile 1 (0–27)	1.00 (ref)	
Quartile 2 (27.01–48)	0.63	0.10,3.84
Quartile 3 (48.01–84)	1.98	0.49, 8.11
Quartile 4 (≥84.01)	1.44	0.32, 6.57
Morning stiffness	2.80	0.22, 36.39
Hip pain or stiffness	0.42	0.15, 1.23
Prednisolone dose per s.d.^a^^,^^b^	0.91	0.55, 1.50
Ever smoker	0.48	0.19, 1.24

ref: reference.

Significant associations are in bold.

a s.d.: age 6.7 years, ESR 24.3 mm/h, prednisolone dose 8.9 mg.

b Daily dosage (mg) of prednisolone prescribed at the onset of PMR.

**Table 5.** Relation between characteristics at PMR diagnosis and time to GCA^a^: Cox regression

Variables	HR	95% CI
Symptom ≥30 days at PMR diagnosis	0.45	0.16, 1.30
Age at PMR diagnosis per s.d.^b^	1.21	0.76, 1.93
Female	2.43	0.81, 7.28
ESR at PMR diagnosis per s.d.^b^	1.10	0.68, 1.70
CRP at PMR diagnosis (mg/l)		
Quartile 1 (0–27)	1.00 (ref)	
Quartile 2 (27.01–48)	0.55	0.10, 3.45
Quartile 3 (48.01–84)	1.76	0.44, 7.10
Quartile 4 (≥84.01)	1.24	0.27, 5.56
Morning stiffness	2.62	0.12, 32.36
Hip pain or stiffness	0.43	0.15, 1.25
Prednisolone dose per s.d.^b^^,^^c^	0.89	0.55, 1.44
Ever smoker	0.50	0.20, 1.26

a GCA diagnosis >1 month after PMR diagnosis.

b s.d.: age 6.7 years, ESR 24.5 mm/h, prednisolone dose 8.9 mg.

c Daily dosage (mg) of prednisolone prescribed at the onset of PMR.

A tendency towards a lower risk for GCA in patients with PMR who had ever smoked was observed [OR 0.48 (95% CI 0.19, 1.24) for GCA >1 month after the PMR diagnosis and for GCA at the PMR diagnosis or later [OR 0.65 (95% CI 0.36, 1.18)].

## Discussion

In this cohort of 626 patients with verified PMR diagnosed in primary or in specialist outpatient or inpatient care, a structured review of medical records demonstrated that 9% developed GCA—6% at or within 1 month of the PMR diagnosis. An additional 3% (*n* = 20) developed GCA >1 month after the PMR diagnosis. Female sex was significantly associated with GCA at the PMR diagnosis or any time after. There was a similar trend in those developing GCA >1 month after the PMR diagnosis, although non-significant, possibly due to the limited sample size. These findings could possibly indicate that the previously described female predominance is a feature of patients with GCA with or without PMR rather than of patients with PMR overall. A female overrepresentation has been seen in several other autoimmune rheumatic conditions, including SLE [29], RA [30] and SS [31]. Findings from studies on hormone-related risk factors (e.g. early menopause and history of breastfeeding) for GCA are contradictory [32, 33].

The starting dose of prednisolone at the PMR diagnosis was significantly higher in patients with GCA at or any time after PMR diagnosis but not in patients who developed GCA >1 month after PMR, likely reflecting an initial clinical suspicion for GCA with other indicating features such as elevated ESR that led to an initial treatment with higher doses of glucocorticosteroids.

Unlike patients with GCA, who are often followed in a rheumatology clinic, PMR is typically managed in primary healthcare. Patients with GCA are at risk of severe complications, including permanent loss of vision, and early initiation of treatment is therefore crucial. Improved knowledge of predictors of GCA in patients with PMR could be valuable for identifying individuals at a higher risk for GCA. The findings of this study indicate a higher risk for GCA in female patients with PMR. Further, a tendency was seen towards a lower risk for GCA in those who had ever smoked. In studies on risk factors for GCA in the general population, a significant association was seen with higher levels of the high-density lipoprotein component ApoA-I [34] and with lower fasting blood glucose, cholesterol and triglyceride levels at baseline after adjustment for current smoking [35]. Both PMR and GCA have been described to have similar cytokine signalling of systemic inflammation [5, 6], although more defined features of autoimmune disease are seen in GCA, with the differentiation of Th-1 cells that drive the chronic disease process [7, 8]. Our study suggests some differences in baseline features between patients with PMR who develop GCA and those who do not, and it could be interesting to investigate whether they also have a different cytokine signalling profile.

Our results on a link between systemic inflammation and GCA in patients with PMR is in agreement with other studies. For example, in a hospital-based study from northern Spain, patients with GCA and PMR were found to have higher ESR levels and platelet counts and lower haemoglobin compared with those with isolated PMR [36].

Screening for subclinical vasculitis in patients with clinical PMR and features of severe systemic inflammation has been proposed. A method that has been used for an extensive period of time in Lugo, Spain, is to perform TABs in cases with PMR and ESR >80 mm/h or constitutional symptoms [37]. In the present study setting, there was no established routine for such screening and indications for TABs and other investigations may have varied over time and across care providers.

In a series of patients with established clinical PMR and no cranial features of GCA undergoing PET/CT scans due to refractory disease or unusual manifestations, increased uptake in the aorta and its branches was seen more often in patients with lower extremity pain, pelvic girdle pain or low back pain [38]. This could partly reflect ischaemia in patients with large vessel involvement. These findings contrast with our study, where hip pain/stiffness at diagnosis was associated with a reduced risk of GCA. As the majority of patients diagnosed with GCA in our study presented with cranial symptoms, we cannot exclude that the relation to such manifestations of musculoskeletal inflammation may be different for aortic involvement compared with cranial GCA.

Limitations of the study are related to the retrospective design. The patients were managed according to clinical practice and not systematically examined according to a structured protocol. Verification of the PMR diagnosis was based on expert review of the medical records. The availability of data on classification criteria items was variable, but almost all patients fulfilled one or more criteria sets. Furthermore, data from primary healthcare by private providers is not included due to lack of access. Finally, the study is based on information from southern Sweden and may not be generalizable to other ethnic populations and other healthcare systems.

Strengths of the study include the large total sample size that underwent a structured review and the inclusion of data from primary care. Every available medical record was reviewed, without any selection. The study was performed in a geographic area where PMR and GCA are commonly occurring and there is therefore reason to believe that the clinical features captured represent a relevant phenotype.

In conclusion, PMR is an inflammatory condition that is typically managed in primary care. A limited proportion of the patients with PMR also develops GCA with the risk of severe complications. In this study of patients with verified PMR, women had a >2-fold increased risk for GCA. A lower risk was seen in those who presented with pain/stiffness in the hip. The findings could possibly reflect different ends of a PMR–GCA spectrum, with more proximal musculoskeletal symptoms and not as strong female overrepresentation in isolated PMR when compared with those with PMR and clinical GCA. Additional studies are indicated to further clarify the connection between PMR and GCA and to identify patients with PMR at higher risk for GCA who need risk-stratified management.

## Data Availability

A limited dataset containing the data that support the main analyses is available from the corresponding author upon reasonable request.
